# Supplementary material for: Sympathetic nervous system controls resolution of inflammation via regulation of repulsive guidance molecule A
Source: Nat Commun. 2019 Feb 7;10:633. doi: 10.1038/s41467-019-08328-5 (PMC6367413; doi:10.1038/s41467-019-08328-5)
Supplement: Supplementary file 1 — Supplementary Information [file 41467_2019_8328_MOESM1_ESM.pdf]

Sympathetic Nervous System Controls Resolution of Inflammation via Regulation of Repulsive Guidance Molecule A

Körner et. al.

Supplementary Information

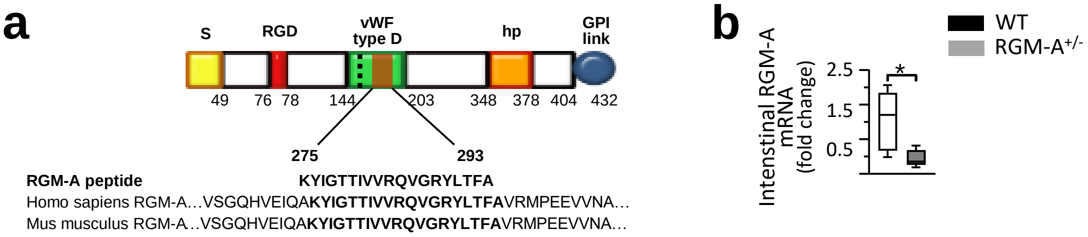

Supplementary Figure 1: a) Schematic representation of the RGM-A region, peptide (aa 284-293), required for binding to its receptor neogenin[2]. **b)** Real time RT-PCR analysis of the RGM-A mRNA expression in the colon of RGM-A$^{+/+}$ and RGM-A$^{+/-}$ mice**.** The following primers were used: *mu RGM-A*: 5'- CTT CCC CGC AGC CAT CT - 3', 5'- CCT CTA TGC CAT GGA CAG CC - 3'. *mu GAPDH*: 5'- ACA TCA AGA AGG TGG TGA AGC - 3', 5'- AAG GTG GAA GAG TGG GAG TG - 3'. Data are representative of two experiments with n=5 mice per group and are expressed as the median ± 95% CI, *P<0.05, unpaired two tailed Student's t-test.

Suppl. Figure 1

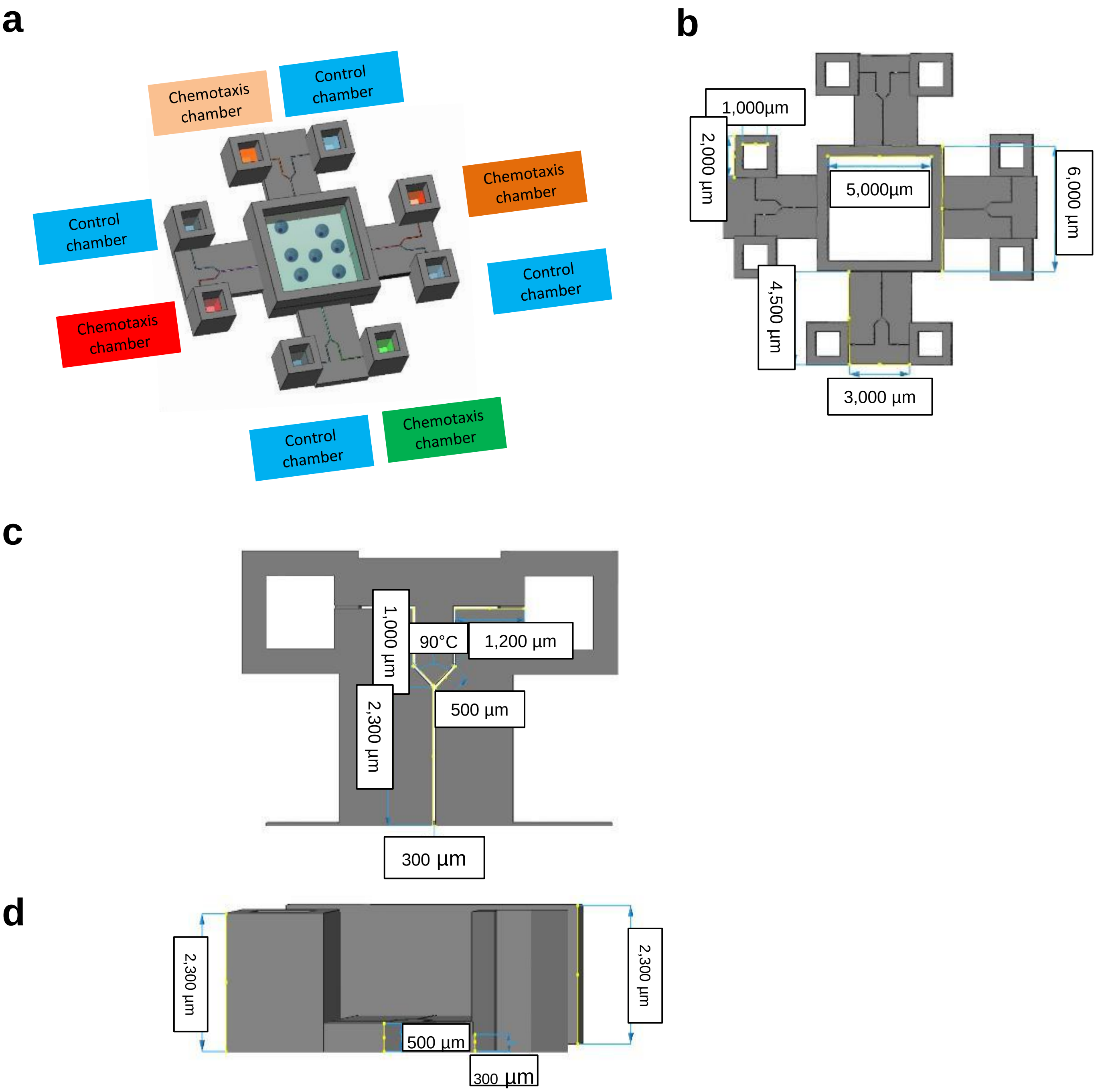

Supplementary Figure 2: Large-scale scheme and fabrication of the microfluidic device. (Magnification 5×). Micro fluidic devices containing eight peripheral and one central cell-loading chamber were printed using an S30L DLP printer (Rapidshape, Heimsheim, Germany) with photoresist MP300 (Rapidshape) according to the manufacturer's instructions. The device was constructed using Netfabb Professional 5.2 (Netfabb, Lupburg, Germany). **Chemotaxis and chemokinesis measurement:** PMNs or MΦ were stained with 10 µl of 0,05% rhodamine-6G (per 1 ml of medium; Sigma-Aldrich) according to the manufacturer's instructions. First, to ensure fluidic communication between all channels, the peripheral chemotaxis chambers were loaded with 1 µl of RPMI and the central cell-loading chamber was loaded with 25 µl of RPMI. To establish the chemoattractive gradients, the chemotaxis chambers were loaded with fMLP (1 µl; Sigma-Aldrich), MCP-1 (1 µl; R&D Systems) or the RGM-A peptide (1 µl), whereas the control chambers were loaded with RPMI to assess the impact on the chemokinesis response. Ten microliters of cell solution (containing 5x10^4 cells) was added to the central cell-loading chamber. The cells were incubated at 37° C for either 2 h (neutrophils) or 8 h (macrophages) prior to imaging the cell migration with an LSM 510 Meta fluorescence microscope (Carl Zeiss, Jena, Germany) (100× magnification) and enumerating the recruited cells using a Casy TT cell counter (Omni Life Science, Bremen, Germany).

Suppl. Figure 2

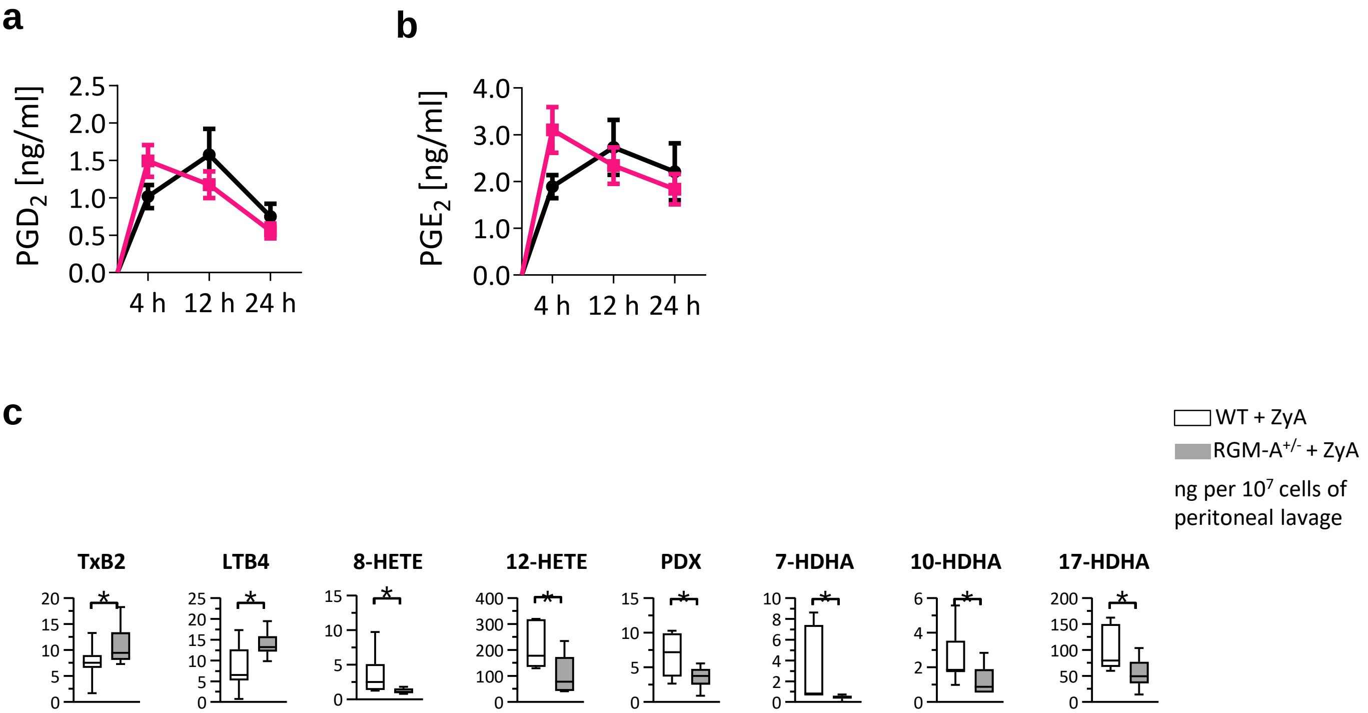

Supplementary Figure 3: RGM-A induces lipid mediator class-switching. a-b) C57BL/6 mice were injected with ZyA and subsequently with either vehicle or a RGM-A peptide; lavages were collected at 4, 12, and 24 h, and LC-MS/MS-based profiling was performed. Levels of Prostaglandin D_2 (PGD_2) **a)** und E_2 (PGE_2) **b)**. The results represent three independent experiments with n=4-17 mice/group and are expressed as the mean±SEM. All results are reported as ng/ml. **c)** Heterozygous RGM-A-deficient (RGM-$A^{+/-}$) mice and their littermate controls were injected with ZyA, and peritoneal lavages were collected at 4 h. Levels of bioactive lipid mediators and precursors including the arachidonic acid (AA), docosahexanoic acid (DHA) and eicosapentaenoic acid (EPA) pathway were quantified by LC-MS/MS-based profiling The results represent two independent experiments and are expressed as mean±SEM (a, b, n=15) or median±95% CI (c, n=8), *P<0.05, unpaired two tailed Student's t-test.

Suppl. Figure 3

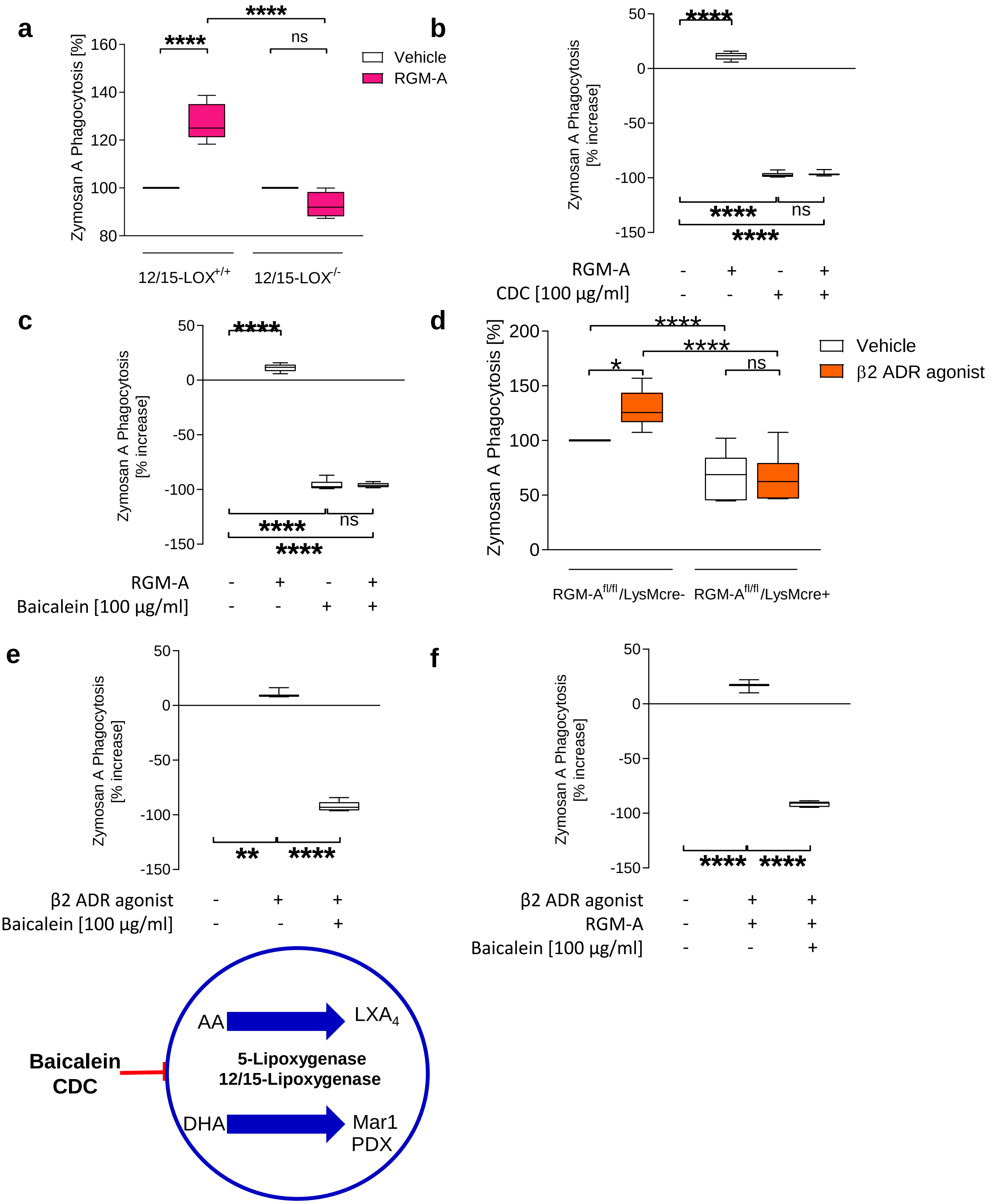

Supplementary Figure 4: Pro-resolving effects of RGM-A and β2 ADR agonists are LOX dependent. MΦ from 12/15-LOX $^{-/-}$ mice **a)** or RGM-A$^{fl/fl}$/LysMcre+ mice **d)** and littermate controls were stimulated either with RGM-A or β2 ADR agonist and vehicle and phagocytosis of fluorescently labeled ZyA particles was determined. Human MΦ were stimulated with RGM-A and the LOX-inhibitors cinnamyl-3,4-dihydroxy-α-cyanocinnamate (CDC) **b)** or baicalein **c)**. In a separate experiment, human MΦ were stimulated with $β_2$ ADR agonist alone **e)** or in combination with RGM-A **f)** and LOX-inhibitor baicalein. The results are from one representative experiment **e-f)** or represent two independent experiments and are expressed as median±95% CI, a, e, f: n=5, b, c: n=10, d: n=6, *P<0.05, **P<0.01; ***P<0.001; ***P<0.0001, ****P<0.00001, One-way ANOVA with Bonferroni correction.

Suppl. Figure 4

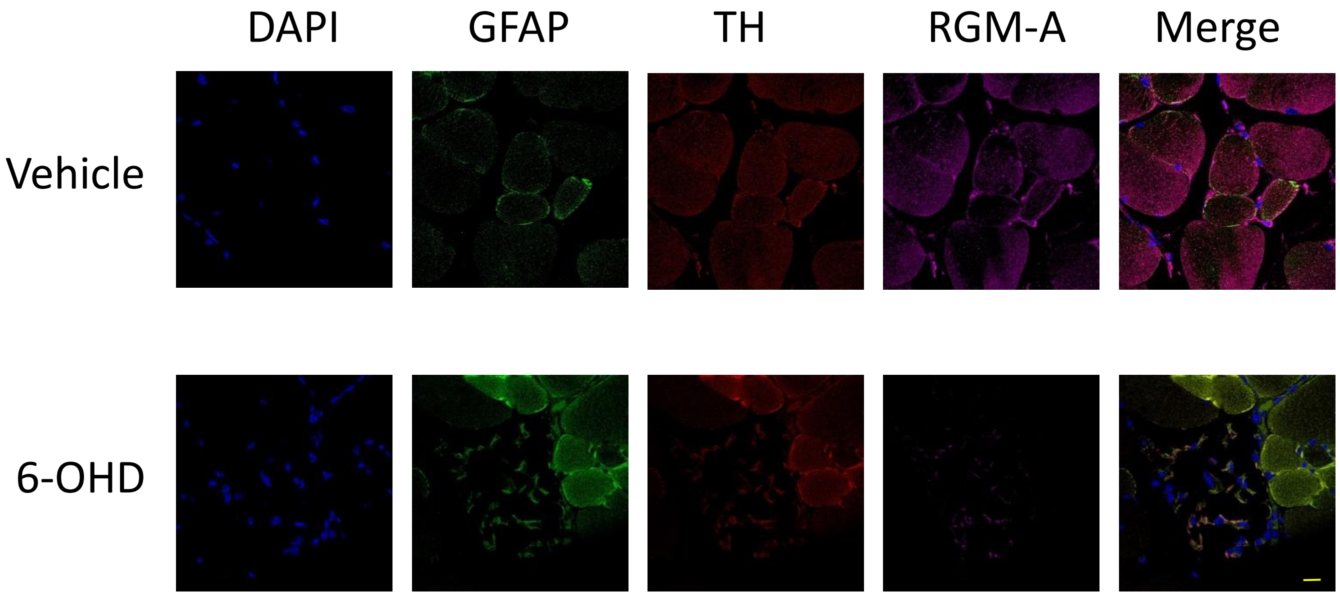

Supplementary Figure 5: Chemical sympathectomy suppresses RGM-A expression. WT animals_were injected with ZyA and vehicle or 6-hydroxydopamine (6-OHD) for 4 h. The expression of RGM-A within the neurofilament structures of peritoneum was analyzed by immunofluorescence, scale bar indicates 40 µm.

Suppl. Figure 5

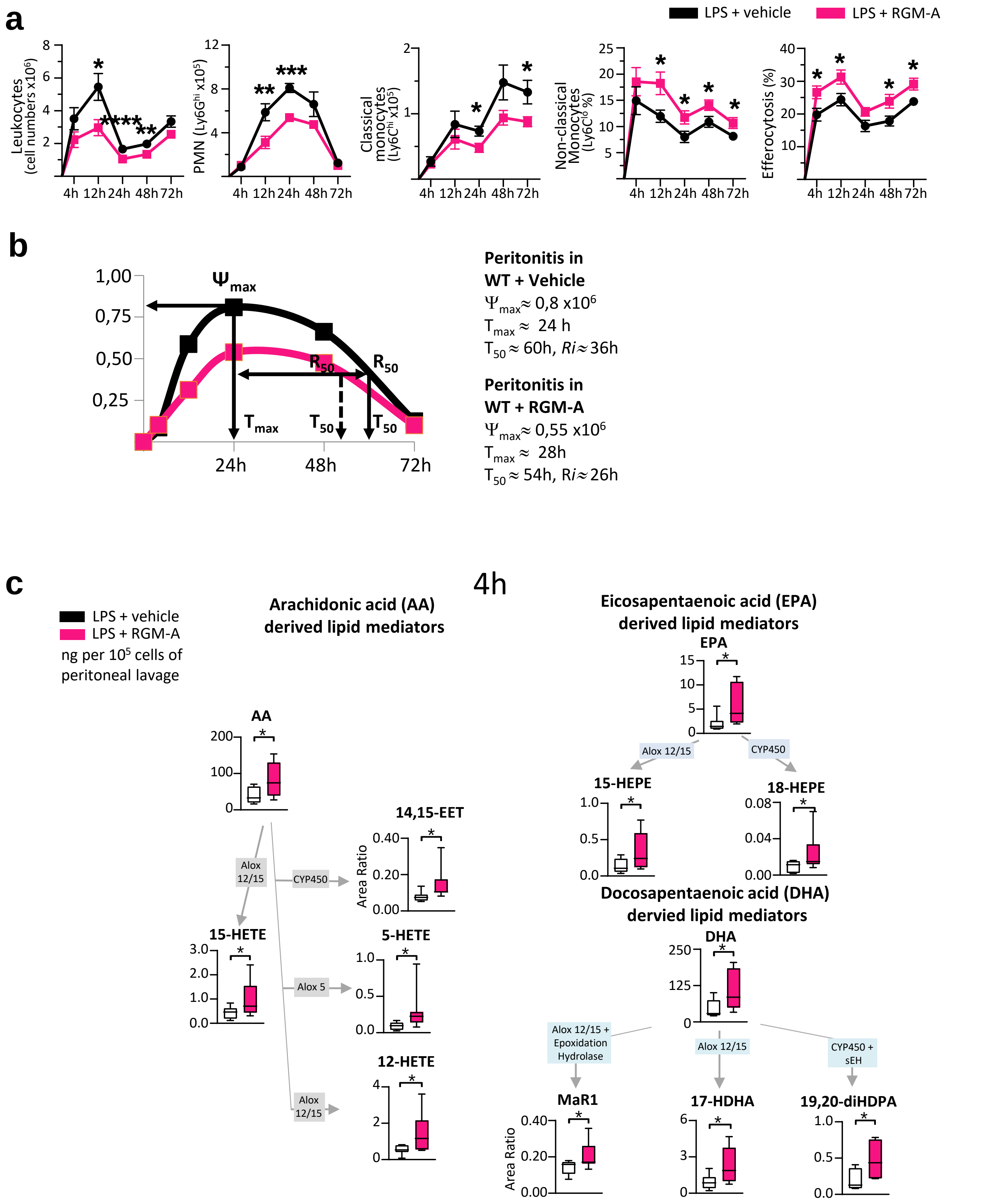

Suppl. Figure 6

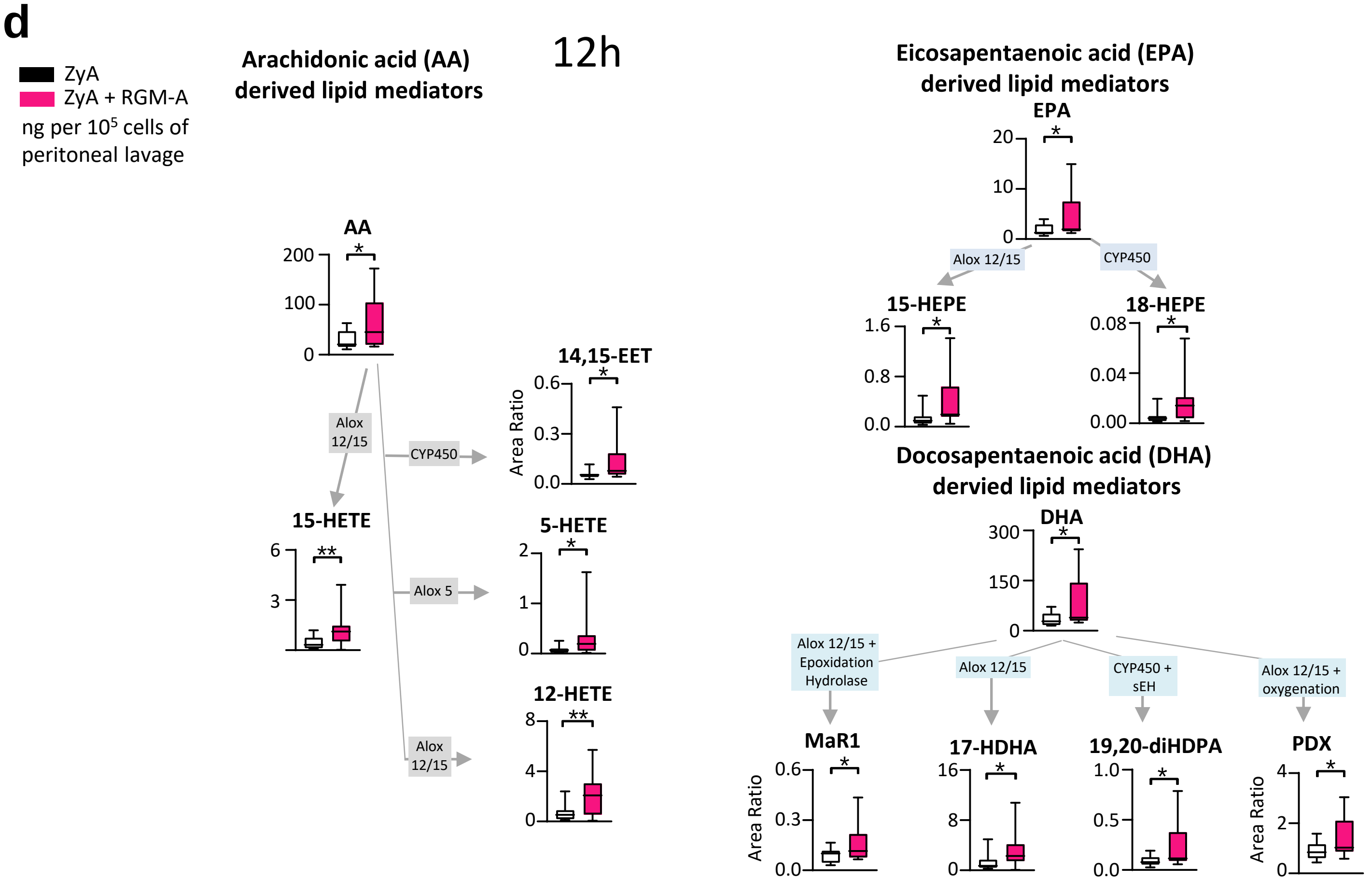

Supplementary Figure 6: Administration of RGM-A dampens acute inflammation and enhances resolution during LPS-induced peritonitis. C57BL/6 mice were injected i.p. with LPS and subsequently i.v. with vehicle or RGM-A. Peritoneal lavages were collected at 4, 12, 24 and 48 hours. Total leukocytes were enumerated by light microscopy, PMN, classical-, non-classical monocytes, peritoneal macrophages and monocyte-derived macrophage efferocytosis were determined by flow cytometry **a).** Resolution indices were calculated as defined by [3] **b)**. For lipidomics, peritoneal fluids were collected and analyzed using LC-MS/MS at 4 hours **c)** and 12 hours **d)**. The results represent two independent experiments and are expressed as mean±SEM (a) or median±95% CI (c, d), n=10 per group, **P<0.05; **P<0.01; ***P<0.001; ***P<0.0001, unpaired two tailed Student's t-test.

a

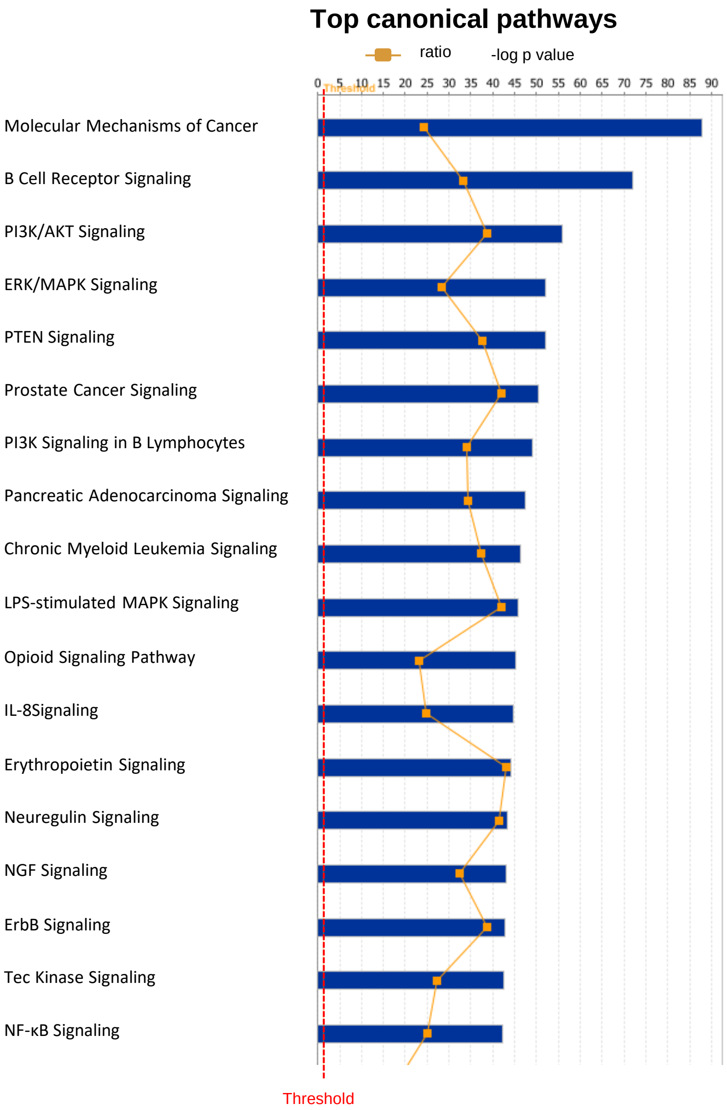

b

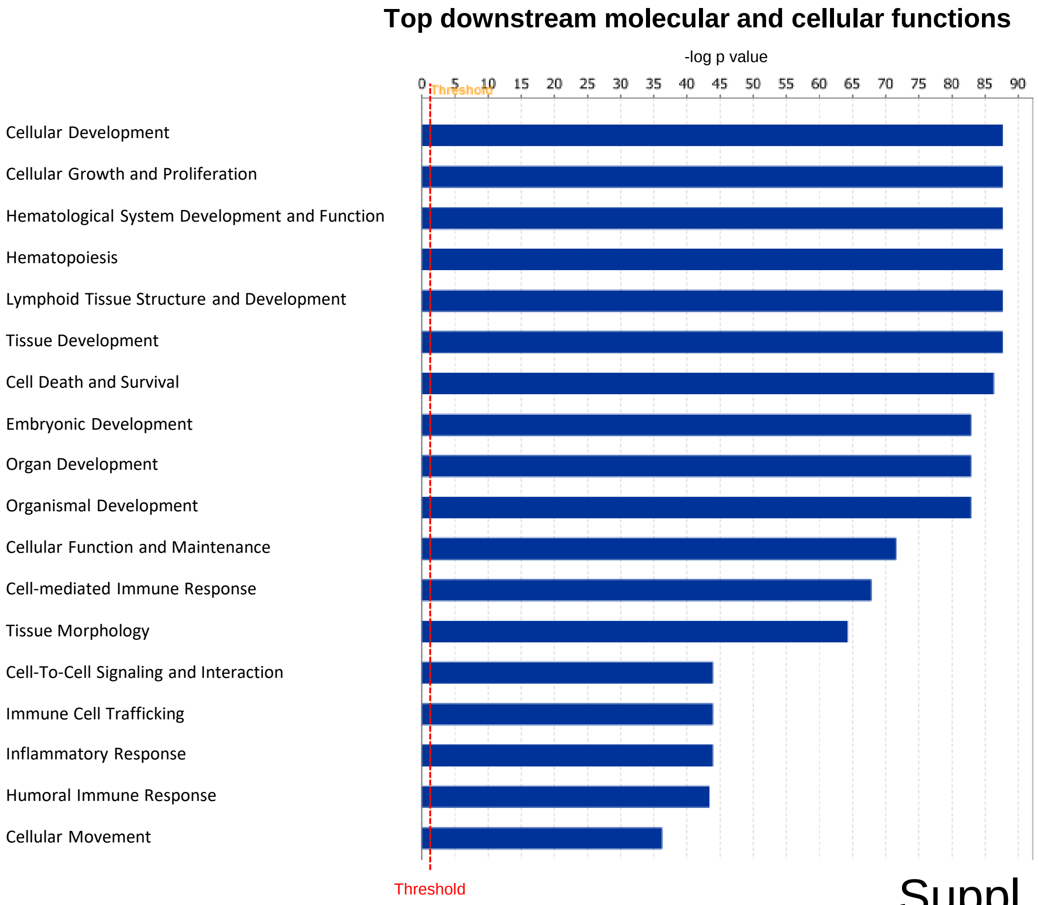

Suppl. Figure 7

c

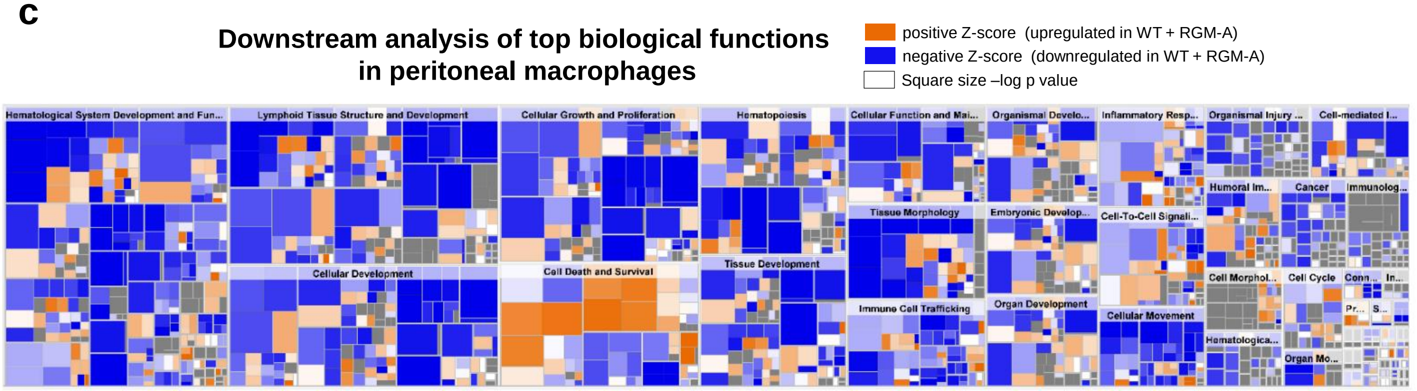

d

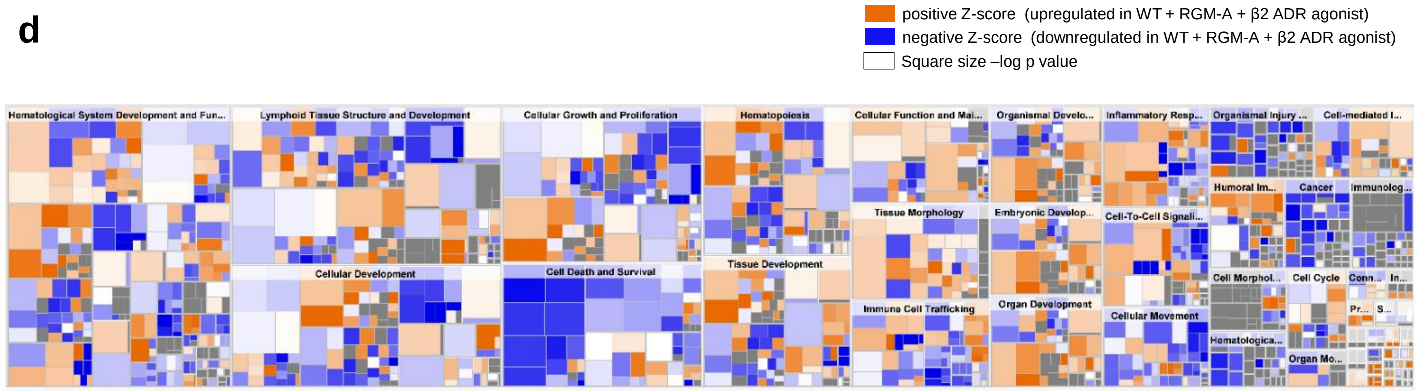

Supplementary Figure 7: Canonical pathways, top function and networks derived from protein profiling of murine peritoneal monocytes of WT mice exposed to ZyA peritonitis and subsequent stimulated with either RGM-A peptide alone and with β2AR agonist. Peritoneal lavages mice were collected 12 h after ZyA treatment and protein expression and phosphorylation were measured in peritoneal monocytes by using a protein microarray. **a-d)** Top molecular and cellular functions regulated in peritoneal monocytes of WT mice treated with or without RGM-A. Most relevant canonical pathways of differentially regulated proteins in WT peritoneal monocytes treated with or without RGM-A. Ratio calculated by the number of measured proteins compared with the total number of proteins involved in the pathway. Downstream effect analysis of biological functions regulated in WT mice with RGM-A- or RGM-A and β2AR agonist stimulation and littermates. Peritoneal monocytes from 4 mice/condition were pooled for analysis. The -log p value was calculated by the Fisher’s exact test right-tailed.

Suppl. Figure 7

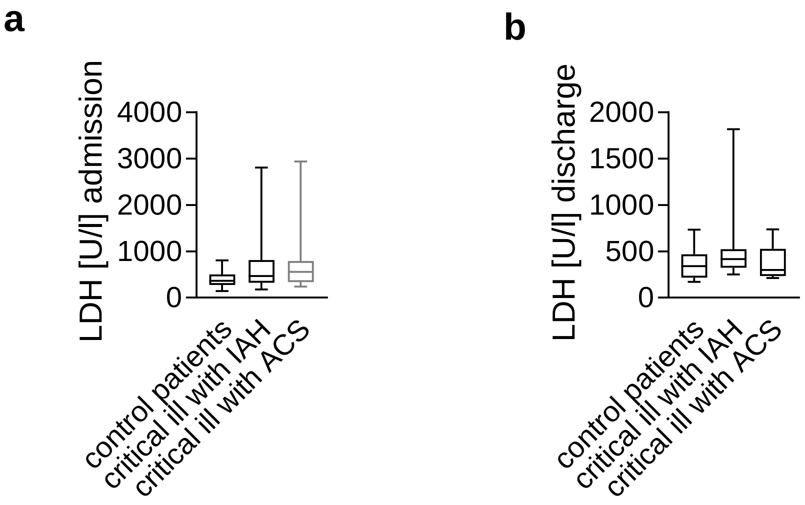

Supplementary Figure 8: Lactate dehydrogenase (LDH) levels (U/L) of study patients. LDH was measured from PICU patients with and without ACS within 24 h after admission (n=85) **a)** and on day of discharge (n=56) **b)** from interdisciplinary PICU. Results are displayed as median±95% CI, non-parametric Kruskal-Wallis test followed by Dunns post-hoc test.

Suppl. Figure 8

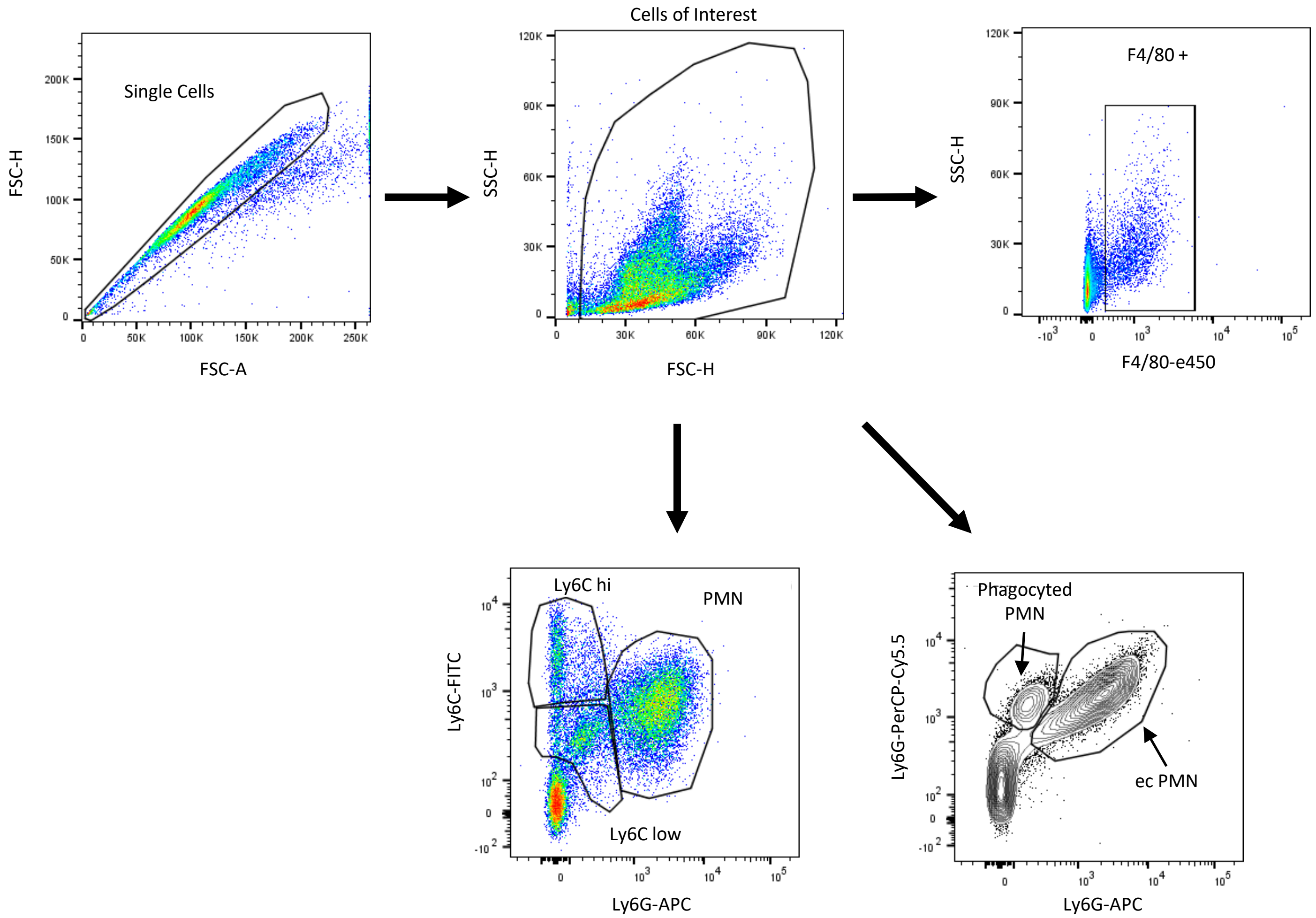

Supplementary Figure 9: FACS gating strategy for leukocyte differentiation and efferocytosis. Leukocytes were gated on FSC/SSC. Leukocyte subtypes were further classified into Ly6G^{hi}, Ly6C^{hi} and Ly6C^{lo}. For defining efferocytosis, the differentiation of intra- and extracellular PMN was assessed by using Ly6G-PerCP-Cy5.5 and Ly6G-APC antibodies. Phagocytized PMNs were Ly6G-PerCP-Cy5.5 positive (+) and Ly6G-APC negative (-).

Common name	Lipidmaps ID	WT + ZyA n=18		WT + ZyA + RGM-A n=6			WT + ZyA + Formoterol n=6			WT + ZyA + RGM-A + Formoterol n=6		
		Mean	SEM	Mean	SEM	P value	Mean	SEM	P value	Mean	SEM	P value
LA	LMFA01030120	7387	704	11 110	963	0.0046 **	13 427	1361	0.0004 ***	20220	5399	0.0016 **
**Arachidonic Acid Bioactive Metabolome**												
AA	LMFA01030001	3273	327	5787	732	0.0048 **	4011	937	0.3504	8389	1627	0.0001 ***
TXB2	LMFA03030002	8.36	1.11	16.73	2.70	0.0065 **	19.56	4.46	0.0013 **	41.62	4.53	<0.0001 ****
PGD$_2$	LMFA03010004	3.22	0.45	7.09	0.83	0.0002 ***	5.57	1.14	0.0410 *	12.42	1.72	<0.0001 ****
PGE$_2$	LMFA03010003	6.46	1.10	13.48	1.73	0.0015 **	11.67	2.45	0.0439 *	32.01	4.17	<0.0001 ****
5-HETE	LMFA03060002	22.64	4.06	49.41	7.87	0.0039 **	18.98	5.61	0.6876	73.02	15.06	<0.0001 ****
LTB$_4$	LMFA03020001	21.38	6.95	61.49	17.56	0.0464 *	59.00	15.90	0.0297 *	165.90	34.72	<0.0001 ****
6-trans-LTB$_4$	LMFA03020013	5.57	1.55	15.08	4.02	0.0428 *	12.94	3.48	0.0359 *	40.10	7.82	<0.0001 ****
6t.12epi-LTB$_4$	LMFA03020014	4.57	0.85	10.22	1.88	0.0117 *	8.55	1.88	0.0428 *	19.12	3.14	<0.0001 ****
8-HETE	LMFA03060006	0.94	0.12	5.03	1.87	0.0435 *	1.50	0.21	0.0300 *	2.60	0.53	0.0003 ***
11-HETE	LMFA03060003	7.28	1.31	17.43	3.87	0.0184 *	7.98	1.31	0.7741	23.55	4.19	<0.0001 ****
0.812-HETE	LMFA03060007	14.69	1.88	35.33	7.87	0.0194 *	50.24	6.25	<0.0001 ****	62.88	9.15	<0.0001 ****
15-HETE	LMFA03060001	6.99	1.06	35.25	9.00	0.0071 **	14.24	1.19	0.0016 **	21.08	2.07	<0.0001 ****
LXA$_4$	LMFA03040001	0.23	0.04	0.52	0.09	0.0022 **	0.25	0.05	0.7480	0.61	0.06	0.0001 ***
**Eicosapentaenoic Acid Bioactive Metabolome**												
EPA	LMFA01030759	193.10	22.58	300.20	42.83	0.0403 *	198.10	34.65	0.9079	451.40	87.35	0.0012 **
15-HEPE	LMFA03070009	1.50	0.23	2.72	0.50	0.0303 *	2.78	0.21	0.0056 **	3.32	0.34	0.0005 ***
18-HEPE	LMFA03070038	0.48	0.07	1.71	0.44	0.0103 *	0.37	0.03	0.4780	0.93	0.13	0.0042 **
14,15-diHETE	LMFA03060077	2.85	0.48	6.98	1.44	0.0125 *	7.47	0.80	0.0002 ***	22.16	3.56	<0.0001 ****
**Docosahexaenoic Acid Bioactive Metabolome**												
DHA	LMFA01030185	3641	508	5247	882	0.1181	4195	902	0.6128	10330	2074	<0.0001 ****
PDX	LMFA04000047	1.48	0.37	5.73	1.96	0.0441 *	0.74	0.08	0.2959	0.97	0.15	0.5105
MaR1	LMFA04050001	0.18	0.02	0.33	0.07	0.0358 *	0.76	0.20	0.0002 ***	0.67	0.08	<0.0001 ****
19,20-diHDPA	LMFA04000043	15.59	2.61	30.66	6.36	0.0373 *	19.36	4.02	0.5183	43.96	7.25	0.0001 ***
10-HDHA	LMFA04000027	1.78	0.42	4.09	1.49	0.1545	4.00	0.29	0.0070 **	4.44	0.81	0.0054 **
7-HDHA	LMFA04000025	0.63	0.11	1.47	0.36	0.0420 *	1.27	0.35	0.0346 *	4.68	1.35	0.0001 ***
17-HDHA	LMFA04000072	20.67	4.06	62.30	18.03	0.0372 *	49.12	3.79	0.0012 **	58.96	6.22	<0.0001 ****

Suppl. Table 1

Supplementary Table 1: Lipid mediator levels in murine peritoneal fluids following administration of ZyA with RGM-A and/or Formoterol in WT mice. Lavages were collected after 4 h and LC-MS/MS-based profiling was performed. Levels of bioactive lipid mediators and precursors derived from the AA, DHA and EPA pathways. All results are reported as ng per 10^7 peritoneal cells. Results represent three independent experiments with n=6-18 mice/group (exact number stated in table) and are expressed as the mean±SEM, unpaired two tailed Student's t-test, *P<0.05; **P<0.01; ***P<0.001; ****P<0.0001 compared to the WT + ZyA group.

		12h					24h				
Common name	Lipidmaps ID	WT + ZyA n=12		WT + ZyA + RGM-A n=8			WT + ZyA n=6		WT + ZyA + RGM-A n=4		
		Mean	SEM	Mean	SEM	P value	Mean	SEM	Mean	SEM	P value
LA	LMFA01030120	5158	586	12278	502	<0.0001 ****	12702	2766	18688	3924	0.2258
**Arachidonic Acid Bioactive Metabolome**											
AA	LMFA01030001	615	148	542	44	0.7912	1282	378	647	92	0.2225
TXB2	LMFA03030002	2.53	0.78	1.80	0.12	0.4771	10.35	2.56	10.91	3.65	0.8997
PGD$_2$	LMFA03010004	0.85	0.17	1.83	0.35	0.0119 *	2.19	1.07	0.72	0.18	0.3085
PGE$_2$	LMFA03010003	1.89	0.37	3.72	0.66	0.0171 *	2.87	0.98	2.10	0.36	0.5592
5-HETE	LMFA03060002	5.31	1.71	14.40	1.22	0.0010 **	1.51	0.46	1.24	0.54	0.7246
LTB$_4$	LMFA03020001	0.7279	0.15	1.33	0.10	0.0094 **	0.12	0.03	0.09	0.03	0.5455
6-trans-LTB$_4$	LMFA03020013	0.43	0.11	0.68	0.05	0.1047	0.17	0.03	0.10	0.03	0.1573
6t.12epi-LTB$_4$	LMFA03020014	0.31	0.05	0.45	0.06	0.0934	0.15	0.03	0.08	0.01	0.1454
8-HETE	LMFA03060006	1.43	0.56	4.40	0.62	0.0026 **	0.49	0.20	0.11	0.03	0.1676
11-HETE	LMFA03060003	2.38	0.80	6.16	0.96	0.0078 **	2.22	0.89	0.80	0.06	0.2945
12-HETE	LMFA03060007	7.31	2.33	10.65	1.22	0.3305	8.13	3.43	1.55	0.81	0.2173
15-HETE	LMFA03060001	4.33	1.83	13.63	2.21	0.0046 **	2.08	0.84	0.73	0.06	0.2931
LXA$_4$	LMFA03040001	0.86	0.36	4.27	1.04	0.0021 **	0.06	0.02	0.07	0.01	0.6471
**Eicosapentaenoic Acid Bioactive Metabolome**											
EPA	LMFA01030759	126.8	40.3	98.4	31.6	0.6416	154.7	65.2	47.3	6.5	0.2272
15-HEPE	LMFA03070009	0.44	0.12	0.71	0.06	0.1134	1.26	0.68	0.30	0.13	0.2969
18-HEPE	LMFA03070038	0.52	0.23	0.97	0.18	0.1719	0.09	0.03	0.05	0.01	0.2389
14,15-diHETE	LMFA03060077	0.24	0.06	0.50	0.10	0.0322 *	0.17	0.06	0.08	0.02	0.3545
**Docosahexaenoic Acid Bioactive Metabolome**											
DHA	LMFA01030185	433.2	92.5	532.2	164.8	0.5765	535.4	136.2	267.4	29.7	0.1575
PDX	LMFA04000047	0.21	0.07	0.38	0.06	0.0888	0.26	0.12	0.05	0.02	0.1991
MaR1	LMFA04050001	0.06	0.02	0.09	0.02	0.2225					
19,20-diHDPA	LMFA04000043	1.02	0.24	2.81	0.49	0.0020 **	0.83	0.23	0.46	0.08	0.2408
10-HDHA	LMFA04000027	0.51	0.16	1.51	0.17	0.0009 ***	0.16	0.06	0.06	0.01	0.2063
7-HDHA	LMFA04000025	0.41	0.20	1.39	0.27	0.0085 **	0.09	0.02	0.05	0.01	0.2770
17-HDHA	LMFA04000072	5.70	1.88	10.53	1.34	0.0820	4.41	2.21	1.07	0.44	0.2681

Suppl. Table 2

Supplementary Table 2: Lipid mediator levels in murine peritoneal fluids following administration of ZyA or ZyA + RGM-A peptide in WT mice. Lavages were collected at 12 and 24 h post ZyA injections and LC-MS/MS-based profiling was performed. All results are reported as ng per10^7 peritoneal cells. Results represent three independent experiments with n=4-12 mice per group (exact number stated in table) and are expressed as the mean±SEM, unpaired two tailed Student's t-test, *P<0.05; **P<0.01; ***P<0.001; ***P<0.001.

Common name	Lipidmaps ID	WT + ZyA+vehicle		WT + ZyA + 6-OHD			WT + ZyA 6-OHD+ RGM-A		
		Mean	SEM	Mean	SEM	P value	Mean	SEM	P value
LA	LMFA01030120	13002	2475	6731	1004	0.0387 *	13467	4832	0.1694
**Arachidonic Acid Bioactive Metabolome**									
AA	LMFA01030001	5114	916	2637	426	0.307 *	4328	899	0.0957
TXB2	LMFA03030002	20.89	3.09	21.26	4.09	0.9425	57.50	23.15	0.1226
PGD_2	LMFA03010004	7.14	1.11	3.71	0.51	0.0150 *	8.74	2.34	0.0148 *
PGE_2	LMFA03010003	12.79	1.95	6.40	0.77	0.0092 **	19.11	6.22	0.0373 *
5-HETE	LMFA03060002	26.26	6.51	5.18	0.63	0.0082 **	10.12	2.10	0.0370 *
LTB_4	LMFA03020001	57.40	14.80	6.35	2.04	0.0047 **	13.78	4.54	0.1394
6-trans-LTB_4	LMFA03020013	15.39	3.14	4.22	0.71	0.0041 **	8.05	2.04	0.0810
6t.12epi-LTB_4	LMFA03020014	10.64	1.93	3.06	0.54	0.0020 **	5.46	1.44	0.1219
8-HETE	LMFA03060006	1.23	0.27	0.48	0.12	0.0396 **	1.69	0.61	0.0686
11-HETE	LMFA03060003	19.18	6.03	8.63	1.31	0.1288	18.14	4.16	0.0349 **
12-HETE	LMFA03060007	36.77	10.43	12.25	3.20	0.0487 *	42.47	14.83	0.0419 **
15-HETE	LMFA03060001	9.94	1.48	5.46	0.88	0.0191 **	10.07	1.66	0.0190 **
LXA_4	LMFA03040001	0.32	0.04	0.15	0.02	0.0027 **	0.27	0.05	0.0387 **
**Eicosapentaenoic Acid Bioactive Metabolome**									
EPA	LMFA01030759	282.5	77.0	181.2	31.2	0.2659	302.3	84.4	0.1788
15-HEPE	LMFA03070009	2.10	0.56	1.49	0.38	0.4118	5.89	3.33	0.2064
18-HEPE	LMFA03070038	0.76	0.34	0.38	0.05	0.3071	0.82	0.32	0.1744
14,15-diHETE	LMFA03060077	4.62	0.85	3.44	0.43	0.2877	310.16	4.38	0.1658
**Docosahexaenoic Acid Bioactive Metabolome**									
DHA	LMFA01030185	6356	1362	3978	532	0.1592	7.386	1394	0.0294 **
PDX	LMFA04000047	2.44	1.37	0.53	0.12	0.3273	2.57	0.82	0.0445 *
MaR1	LMFA04050001	0.91	0.39	0.23	0.08	0.2625	0.48	0.31	0.5374
19,20-diHDPA	LMFA04000043	25.23	3.69	25.76	3.18	0.9160	57.96	14.61	0.0368 *
10-HDHA	LMFA04000027	1.69	1.00	0.49	0.16	0.2836	1.15	0.51	0.2126
7-HDHA	LMFA04000025	1.325	0.49	0.23	0.04	0.0459 *	0.43	0.08	0.0279 *
17-HDHA	LMFA04000072	29.92	9.66	15.07	4.46	0.2026	58.69	30.15	0.1496

Suppl. Table 3

Supplementary Table 3: Lipid mediator levels in murine peritoneal fluids following the chemical sympathectomy with 6-OHD and ZyA peritonitis with vehicle or RGM-A treatment in WT mice. Lavages were collected 4 h post ZyA injections and LC-MS/MS-based profiling was performed. All results are reported as ng per 10^7 peritoneal cells. Results represent three independent experiments with n=10 mice per group and are expressed as the mean±SEM. ZyA + vehicle was compared to ZyA + 6-OHD as well as ZyA + 6-OHD to ZyA + 6-OHD + RGM-A by unpaired two tailed Student's t-test, *P<0.05; **P<0.01; ***P<0.001.

a

Day of admission	RGM-A		S-CRP		Interleukin 6 (IL-6)		Procalcitonin (PCT)		WBC	
	ρ	P-value (n=108)	ρ	P-value (n=92)	ρ	P-value (n=63)	ρ	P-value (n=12)	ρ	P-value (n=88)
RGM-A			0,103	0,333	0,177	0,188	0,720	0,011	-0,162	0,142
LDH	0,261	0,020	0,331	0,006	0,318	0,031	0,467	0,179	0,320	0,009
Lactate [mmol/l]	0,291	0,002	-0,008	0,943	0,330	0,008	0,428	0,166	0,121	0,263
Bilirubin [µmol/l]	0,184	0,091	0,0606	0,618	0,071	0,641	0,503	0,144	0,149	0,198
S-CRP [mg/l]	0,106	0,333			0,045	0,755	0,525	0,082	-0,001	0,991
IL-6 [ng/l]	0,177	0,188	0,0453	0,755			-0,083	0,843	-0,187	0,236
Creatinine [µmol/l]	0,062	0,535	0,248	0,021	0,187	0,149	0,182	0,595	0,341	0,001
PCT [µg/l]	0,720	0,011	0,525	0,082	-0,083	0,843			-0,048	0,935
WBC	-0,162	0,142	-0,001	0,991	-0,187	0,236	-0,048	0,935		
IAH grade	0,236	0,014	0,374	<0,001	0,135	0,295	0,330	0,293	-0,082	0,447
PRISM III	0,287	0,003	0,338	<0,001	0,335	0,008	0,347	0,268	-0,063	0,560

b

Day of discharge	RGM-A		S-CRP		Interleukin 6 (IL-6)		Procalcitonin (PCT)		WBC	
	ρ	P-value (n=91)	ρ	P-value (n=88)	ρ	P-value (n=73)	ρ	P-value (n=3)	ρ	P-value (n=91)
RGM-A			0,125	0,260	0,212	0,085	1	0,333	0,087	0,423
LDH	0,250	0,071	0,271	0,065	0,061	0,703	0,5	1	0,217	0,131
Lactate [mmol/l]	0,026	0,804	0,032	0,765	-0,032	0,788	0,866	0,667	0,101	0,341
Bilirubin [µmol/l]	0,202	0,107	0,230	0,066	-0,1007	0,473	-0,5	1	-0,041	0,741
S-CRP [mg/l]	0,125	0,260			0,374	0,002	0,5	1	0,002	0,985
IL-6 [ng/l]	0,212	0,085	0,374	0,002			0,5	1	0,124	0,309
Creatinine [µmol/l]	0,015	0,888	0,011	0,921	-0,289	0,015	-0,5	1	0,077	0,472
PCT [µg/l]	1	0,333	0,5	1	0,5	1			1	0,333
WBC	0,087	0,423	0,002	0,985	0,124	0,309	1	0,333		
IAH grade	0,113	0,287	0,095	0,381	-0,127	0,289			-0,086	0,416
PRISM III	0,181	0,093	0,286	0,009	0,074	0,551	1	0,333	0,218	0,044

Supplementary Table 4: Correlations of RGM-A and conventional inflammatory parameters with descriptive, organ and outcome parameters. From 109 PICU patients with and without ACS blood samples were drawn within 24 h after admission to interdisciplinary PICU **a)** and on day of discharge **b)**. Non-parametric Spearman's rank correlation coefficient Rho was calculated and is shown with corresponding p-values.

REFERENCES

1. Itokazu T, Fujita Y, Takahashi R, Yamashita T. Identification of the neogenin-binding site on the repulsive guidance molecule A. *PloS one* **7**, e32791 (2012).

2. Bannenberg GL*, et al.* Molecular circuits of resolution: formation and actions of resolvins and protectins. *Journal of immunology* **174**, 4345-4355 (2005).
