# Supplementary material for: Sympathetic nervous system controls resolution of inflammation via regulation of repulsive guidance molecule A
Source: Nat Commun. 2019 Feb 7;10:633. doi: 10.1038/s41467-019-08328-5 (PMC6367413; doi:10.1038/s41467-019-08328-5)
Supplement: Supplementary file 3 — Description of Additional Supplementary Files [file 41467_2019_8328_MOESM3_ESM.pdf]

Description of Additional Supplementary Files

Supplementary Data1: Protein and phosphorylation profile of murine peritoneal monocytes.
