# Supplementary material for: Sympathetic nervous system controls resolution of inflammation via regulation of repulsive guidance molecule A
Source: Nat Commun. 2019 Feb 7;10:633. doi: 10.1038/s41467-019-08328-5 (PMC6367413; doi:10.1038/s41467-019-08328-5)
Supplement: Supplementary file 5 — Reporting Summary [file 41467_2019_8328_MOESM5_ESM.pdf]

nature research

Corresponding author(s): Prof. Valbona Mirakaj

Last updated by author(s): Jan 2, 2019

Reporting Summary

Nature Research wishes to improve the reproducibility of the work that we publish. This form provides structure for consistency and transparency in reporting. For further information on Nature Research policies, see Authors & Referees and the Editorial Policy Checklist.

Statistics

For all statistical analyses, confirm that the following items are present in the figure legend, table legend, main text, or Methods section.

n/a	Confirmed	
☐	☒	The exact sample size (*n*) for each experimental group/condition, given as a discrete number and unit of measurement
☐	☒	A statement on whether measurements were taken from distinct samples or whether the same sample was measured repeatedly
☐	☒	The statistical test(s) used AND whether they are one- or two-sided<br>*Only common tests should be described solely by name; describe more complex techniques in the Methods section.*
☐	☒	A description of all covariates tested
☐	☒	A description of any assumptions or corrections, such as tests of normality and adjustment for multiple comparisons
☐	☒	A full description of the statistical parameters including central tendency (e.g. means) or other basic estimates (e.g. regression coefficient) AND variation (e.g. standard deviation) or associated estimates of uncertainty (e.g. confidence intervals)
☒	☐	For null hypothesis testing, the test statistic (e.g. *F*, *t*, *r*) with confidence intervals, effect sizes, degrees of freedom and *P* value noted<br>*Give P values as exact values whenever suitable.*
☒	☐	For Bayesian analysis, information on the choice of priors and Markov chain Monte Carlo settings
☒	☐	For hierarchical and complex designs, identification of the appropriate level for tests and full reporting of outcomes
☐	☒	Estimates of effect sizes (e.g. Cohen's *d*, Pearson's *r*), indicating how they were calculated

Our web collection on statistics for biologists contains articles on many of the points above.

Software and code

Policy information about availability of computer code

Data collection	BioRad CFX Manager was used for RT-PCR. FACS data collection was performed with BD FACS Diva software (BD Biosciences)
Data analysis	FlowJo V8 was used for FlowCytometry analysis, GraphPad Prism V8 was used for statistical testing. Ingenuity Pathway Analysis (IPA; Qiagen) was used for pathway analysis.

For manuscripts utilizing custom algorithms or software that are central to the research but not yet described in published literature, software must be made available to editors/reviewers. We strongly encourage code deposition in a community repository (e.g. GitHub). See the Nature Research guidelines for submitting code & software for further information.

Data

Policy information about availability of data

All manuscripts must include a data availability statement. This statement should provide the following information, where applicable:

- Accession codes, unique identifiers, or web links for publicly available datasets
- A list of figures that have associated raw data
- A description of any restrictions on data availability

The antibody array data generated and analyzed during the current study are available in the NCBI's Gene Expression Omnibus database45 through GEO Series accession number GSE122569 ([http://www.ncbi.nlm.nih.gov/geo/query/acc.cgi?acc=GSE122569]).

Field-specific reporting

Please select the one below that is the best fit for your research. If you are not sure, read the appropriate sections before making your selection.

☒ Life sciences ☐ Behavioural & social sciences ☐ Ecological, evolutionary & environmental sciences

For a reference copy of the document with all sections, see nature.com/documents/nr-reporting-summary-flat.pdf

Life sciences study design

All studies must disclose on these points even when the disclosure is negative.

Sample size	For in vivo experiments, a minimum sample size of 6 was assumed sufficient.
Data exclusions	No data were excluded
Replication	All results were verified by repeated measurements
Randomization	Mice were randomized to specific treatment groups.
Blinding	Blinding was not relevant because experiments were verified by sufficient number of repeated measurements.

Reporting for specific materials, systems and methods

We require information from authors about some types of materials, experimental systems and methods used in many studies. Here, indicate whether each material, system or method listed is relevant to your study. If you are not sure if a list item applies to your research, read the appropriate section before selecting a response.

Materials & experimental systems

n/a	Involved in the study	
☐	☒	Antibodies
☒	☐	Eukaryotic cell lines
☒	☐	Palaeontology
☐	☒	Animals and other organisms
☐	☒	Human research participants
☐	☒	Clinical data

Methods

n/a	Involved in the study	
☒	☐	ChIP-seq
☐	☒	Flow cytometry
☒	☐	MRI-based neuroimaging

Antibodies

Antibodies used

anti-CD16/CD32 (Clone 93, BioLegend, #101320, 1:50)
anti-mouse APC-Ly6G (Clone 1A8, BioLegend, # 127614, 1:250)
anti-mouse e450-F4/80 (Clone BM8, eBioscience, #48-4801-82, 1:100)
anti-mouse FITC-Ly6C (Clone HK1.4, BioLegend, #128006, 1:250)
anti-mouse PerCP-Cy5.5-conjugated anti-Ly6G (Clone 1A8, BioLegend, #127616, 15:1000)

anti-GFAP A488-labeled (Clone 2E1.E9, Biolegend, # 644704, 1:250)
rabbit anti-tyrosine hydroxylase (abcam, #ab112, 1:1000)
goat anti-NF-M (SantaCruz, #sc-16143, 1:50)
rabbit anti-RGM-A (Abcam, #ab26287, 1:50)
goat anti-RGM-A (SantaCruz, #sc-46481, 1:50)
Alexa Fluor 594-conjugated donkey anti-goat (Invitrogen, #A-11058, 1:100)
Alexa Fluor 546-conjugated goat anti-rabbit (Invitrogen, #A-11010, 1:100)
Alexa Fluor 647-conjugated goat anti-rabbit (Invitrogen, #A-21244, 1:100)
IgG isotype control antibodies (Santa Cruz Biotechnology)

Validation

The antibodies were selected based on the validations by the manufacturer.

Animals and other organisms

Policy information about studies involving animals; ARRIVE guidelines recommended for reporting animal research

Laboratory animals

C57BL/6 mice, male and female, age 6-10 weeks; RGM-A heterozygous mice on a C57BL/6 background, male and female, age from 6 weeks; RGM-A LysM Cre+ mice, male and female, age from 6 weeks; 12/15-LOX-mice male and female, age from 6 weeks.

Wild animals	No wild animals were studied.
Field-collected samples	No field-collected samples were involved.
Ethics oversight	All animal experiments were carried out according to procedures approved by the Institutional Review Board and the Regierungspräsidium Tübingen and comply with all relevant ethical regulations regarding animal research.

Note that full information on the approval of the study protocol must also be provided in the manuscript.

Human research participants

Policy information about studies involving human research participants

Population characteristics	Healthy donors, both gender and various age from the Department of Anesthesia.
Recruitment	Blood was obtained from healthy donors without suffering from an acute infection and without taking any medication.
Ethics oversight	Ethics Review Committee of the Faculty of Medicine, Eberhard Karls University Tübingen (Approval number 351/2013BO2)

Note that full information on the approval of the study protocol must also be provided in the manuscript.

Clinical data

Policy information about clinical studies

All manuscripts should comply with the ICMJE guidelines for publication of clinical research and a completed CONSORT checklist must be included with all submissions.

Clinical trial registration	WHO-ICTRP DRKS00006556
Study protocol	https://www.drks.de/drks_web/navigate.do?navigationId=trial.HTML&TRIAL_ID=DRKS00006556
Data collection	Critically ill children between 0 and 18 years of age were enrolled between January and August 2015 after obtaining informed written consent from the parents or guardians of each child. Plasma samples were taken pediatric ICU patients from the Pediatric Intensive Care Unit (PICU) of Hannover Medical School (MHH, Germany) within 24 h after admission and at day of discharge.
Outcomes	Primary Outcome(s)<br>Correlation of directly measured intra-abdominal pressures with indirect measuring methods (via the stomach and bladder) and assessment in terms of accuracy (goodness of fit), sensitivity and practicality. Using gastric tubes the IAP can be determined continuously; via the bladder or using direct accesses, however, the IAP can be examined only intermittently. The proposal provides for hourly documentation of continuous readings and a two-hourly collection and documentation of discontinuous IAP-values.<br>Secondary Outcome(s)<br>Investigation of the influence of different intra-abdominal pressures on global hemodynamics and microcirculation in critically ill children. In addition to established intensive care Parameters at least three times daily cardiac output and other volumetric parameters will be determined using the ultrasound dilution technique (macrocirculation). Duplex ultrasound studies of the microcirculation in parenchymal organs are also done three times a day, which later can be evaluated and quantied using the PixelFlux software. Using somatic optodes, tissue oxygen saturation in parenchymal organs will be continuously monitored with the help of near-infrared spectroscopy (NIRS). The results of NIRS determination will be compared with central venous saturations at least 4x daily . In addition to established laboratory chemical organ parameters, the Proteins zonulin and citrulline, as well as different fatty acid binding proteins and micro-RNAs are regularly determined to thereby establish new biomarkers for the early detection of IAP-induced organ and tissue damage.

Flow Cytometry

Plots

Confirm that:

☒ The axis labels state the marker and fluorochrome used (e.g. CD4-FITC).

☒ The axis scales are clearly visible. Include numbers along axes only for bottom left plot of group (a 'group' is an analysis of identical markers).

☒ All plots are contour plots with outliers or pseudocolor plots.

☒ A numerical value for number of cells or percentage (with statistics) is provided.

Methodology

Sample preparation	Peritoneal lavages were collected and cells were blocked with mouse anti-CD16/CD32 (BioLegend, #101320, 1:50) antibodies for 10 min at room temperature and then stained with anti-mouse APC-Ly6G (BioLegend, # 127614, 1:250), e450-F4/80 (eBioscience, # 48-4801-82, 1:100) (all from eBioscience) and FITC-Ly6C (BioLegend, #128006, 1:250) (BioLegend) antibodies for 30 min at 4°C. To analyze the MΦ phagocytosis of apoptotic PMNs in vivo, the cells were permeabilized and then stained with

	PerCP-Cy5.5-conjugated anti-Ly6G (BioLegend, #127616, 15:1000) (eBioscience) for 30 min at 4°C
Instrument	The cells were analyzed by flow cytometry (BD FACSCanto II)
Software	FACS Diva software and Flow Jo were used to collect and analyze data.
Cell population abundance	Purity was greater 90%.
Gating strategy	Leukocytes were gated on FSC/SSC. Leukocyte subtypes were further classified into Ly6Ghi, Ly6Chi and Ly6Clow. For defining efferocytosis, the differentiation of intra- and extracellular PMN was assessed by using Ly6G-PerCP-Cy5.5 and Ly6G-APC antibodies. Phagocytized PMNs were Ly6G-PerCP-Cy5.5 positive (+) and Ly6G-APC negative (-).

☒ Tick this box to confirm that a figure exemplifying the gating strategy is provided in the Supplementary Information.
